# A Non-Linear Ototoxic Response to Cisplatin Is Associated with Autophagy and PTEN/PI3K/AKT Signaling in Cochlear Hair Cells and Spiral Ganglion Neurons

**DOI:** 10.3390/biomedicines14102377

**Published:** 2026-10-19

**Authors:** Yingru Li, Miaomiao Han, Cheng Lu, Jingyi Tang, Maoli Duan, Jun Yang, Huiqun Jie, Qiang Song, Jingchun He

**Affiliations:** 1 Department of Otorhinolaryngology-Head & Neck Surgery, Xinhua Hospital, Shanghai Jiaotong University School of Medicine, Shanghai 200092, China; lyr1116@sjtu.edu.cn (Y.L.);; 2 Ear Institute, Shanghai Jiaotong University School of Medicine, Shanghai 200092, China; 3 Shanghai Key Laboratory of Translational Medicine on Ear and Nose Diseases, Shanghai 200092, China; 4 Department of Otolaryngology, Zibo Central Hospital, Zibo 255000, China; 5 Ear Nose and Throat Patient Area, Trauma and Reparative Medicine Theme, Karolinska University Hospital, 171 76 Stockholm, Sweden; 6 Division of Ear, Nose and Throat Diseases, Department of Clinical Science, Intervention and Technology, Karolinska Institutet, 171 77 Stockholm, Sweden

**Keywords:** cisplatin ototoxicity, autophagy, apoptosis, cochlear organotypic cultures, HC, SGN, PTEN/PI3K/AKT

## Abstract

**Background:** Cisplatin-induced ototoxicity is generally considered dose-dependent. However, cellular adaptive responses under severe cisplatin stress remain incompletely understood. This study aimed to investigate the non-linear ototoxic response to cisplatin and explore the potential involvement of autophagy in the survival of cochlear hair cells and spiral ganglion neurons. **Methods:** Cochlear explants from postnatal day 3 rats and HEI-OC1 cells were treated with different concentrations of cisplatin (0–400 μM). HCs and SGNs injury and apoptosis were assessed by immunofluorescence staining, hair cell counting, TUNEL assays, cell viability assays, and Western blotting. PCR arrays were used to characterize neurotransmission- and autophagy-related gene expression, and transmission electron microscopy was performed to examine autophagic structures. Western blotting was further used to evaluate autophagy-related proteins and PTEN/PI3K/AKT signaling. **Results:** Cisplatin induced a non-linear ototoxic response in cochlear explants. Hair cell loss increased from approximately 70% at 10 μM to over 90% at 100 μM, but was markedly reduced at 400 μM. This reduced injury was accompanied by decreased apoptosis, increased LC3-II, decreased P62, and altered PTEN/PI3K/AKT signaling. In contrast, HEI-OC1 cells showed progressively reduced viability with increasing cisplatin concentration, suggesting a model-dependent response. **Conclusions:** High-concentration cisplatin was associated with reduced cochlear injury and enhanced autophagy-related activity in cochlear explants. These findings suggest that autophagy and PTEN/PI3K/AKT signaling are associated with the non-linear response to severe cisplatin stress.

## 1. Introduction

Cisplatin is a highly effective platinum-based chemotherapeutic agent widely used for the treatment of various solid tumors. However, its clinical application is limited by several dose-limiting adverse effects, including nephrotoxicity, neurotoxicity, and particularly ototoxicity, which can result in progressive and irreversible sensorineural hearing loss [1,2,3]. Within the cochlea, hair cells (HCs), spiral ganglion neurons (SGNs), and the stria vascularis are major targets of cisplatin toxicity. Cisplatin can accumulate in cochlear tissues and induce oxidative stress, DNA damage, inflammation, mitochondrial dysfunction, and, ultimately, sensory cell death [4,5,6,7]. Despite extensive efforts to explore otoprotective strategies, effective interventions remain limited, highlighting the need for a better understanding of the cellular responses that determine cochlear survival or injury after cisplatin exposure [8].

Apoptosis is considered a major mechanism of cisplatin-induced cochlear cell death, and multiple anti-apoptotic pathways have, therefore, been investigated as potential therapeutic targets. The modulation of PTEN/PI3K/AKT signaling or downstream apoptotic pathways can attenuate cisplatin-induced cochlear injury [9,10,11]. However, the suppression of apoptosis alone does not fully explain the cellular response to cisplatin, suggesting that additional stress-adaptive mechanisms are involved. In particular, cellular responses under severe cisplatin stress may be more complex than a simple linear concentration–damage relationship.

Autophagy is an evolutionarily conserved process that maintains cellular homeostasis through the degradation and recycling of damaged proteins and organelles. Increasing evidence indicates that autophagy participates in cisplatin-induced ototoxicity. Appropriate activation of autophagic clearance may promote cochlear cell survival, whereas excessive or impaired autophagy may contribute to cellular injury [12,13,14,15]. The PTEN/PI3K/AKT pathway is also closely associated with HC survival and cellular stress responses and has been linked to the regulation of autophagy [16,17]. Although PI3K/AKT signaling generally suppresses autophagy through mTOR, concurrent AKT activation and autophagy may occur under severe cellular stress [18]. Previous concentration–response studies demonstrated that cochlear hair cell injury did not increase proportionally with cisplatin concentration, suggesting a non-linear ototoxic response [19]. In the present study, 400 μM cisplatin was selected to investigate this previously observed response under severe cisplatin stress. However, the mechanisms underlying this phenomenon, including the potential involvement of the apoptosis–autophagy balance, remain unclear.

Therefore, this study aimed to characterize the concentration–response relationship of cisplatin-induced cochlear injury and to investigate the potential roles of apoptosis and autophagy in this process.

## 2. Materials and Methods

**Cell culture**

The HEI-OC1 cells were grown in high-glucose Dulbecco’s modified Eagle’s medium (DMEM) containing 10% fetal bovine serum (FBS, A5670701, Gibco Life Technologies, Carlsbad, CA, USA) and 1% penicillin-streptomycin (C0222, Beyotime, Shanghai, China), with 10% CO_2_ at 33 °C. At 80% confluence, cells were trypsinized with 0.05% trypsin/EDTA (C0202, Beyotime, Shanghai, China) for subculturing.

**Cell viability**

The HEI-OC1 cells (H1663, QuiCell Biotechnology, Shanghai, China) were detached using a 0.05% solution of trypsin/EDTA. Post-detachment, the cells were collected by centrifugation at approximately 160× *g* for 3 min. The cells were then resuspended in a growth medium and seeded into 96-well plates at a density of 5000 cells per well, with each condition triplicated. Following a 24 h incubation period, the growth medium was replaced with fresh medium containing varying concentrations of cisplatin (0, 10, 100, and 400 μM; P4394, Sigma, St. Louis, MO, USA). Cell viability was quantified using a Cell Counting Kit-8 (CCK-8) assay (HY-K0301, MedChemExpress, Monmouth Junction, NJ, USA), according to the manufacturer’s instructions.

**Animal Procedures**

All cochlear organotypic cultures were prepared from postnatal day 3 (P3) Sprague–Dawley rat pups purchased from the Shanghai Laboratory Animal Research Center (Shanghai, China). All experimental procedures were approved by the animal ethics guidelines of the Institutional Animal Care and Use Committee of Shanghai Jiao Tong University (Approval Code: XHEC-NSFC-2022-244). Strict attention was paid to the care and use of animals, according to Chinese Law.

**Cochlear Organotypic Cultures**

Briefly, the P3 rat pups were decapitated, and their cochleae were carefully removed and placed in Hank’s Balanced Salt Solution (G4203, Servicebio, Wuhan, China). The lateral wall was dissected away, and the whole basilar membrane, containing the organ of Corti and spiral ganglion neurons, was isolated from the modiolus of the cochlea for transfer onto the cell culture plate. A drop (5 µL) of cell-tak (35420, Corning, NY, USA) was placed in the center of a 12-well cell culture plate for approximately 20 min at room temperature. Afterward, 2 mL of DMEM/F12 growth medium (11320082, Gibco Life Technologies, Carlsbad, CA, USA), 2% B-27TM supplement (17504044, Gibco Life Technologies, Carlsbad, CA, USA), 1% N-2 supplement (17502001, Gibco Life Technologies, Carlsbad, CA, USA), and ampicillin (50 g/mL, A5354-10ML, Sigma-Aldrich, St. Louis, MO, USA) were added to the 12-well cell culture well. The cochlear explant was cultured at 37 °C in 5% CO_2_ overnight. On the following day, fresh medium was added alone or containing various concentrations of cisplatin [19].

**Cisplatin treatment**

The cisplatin solution was freshly made at a concentration of 1 mM in serum-free medium and diluted to final concentrations of 0, 10, 100, and 400 µM. The concentrations of cisplatin were selected based on our preliminary and previous experiments examining cochlear explants exposed to 10–1000 μM cisplatin for 48 h. These experiments identified substantial cochlear injury at 10–100 μM, but unexpectedly reduced morphological injury at higher concentrations. Therefore, 10 and 100 μM were selected to represent concentrations associated with progressive injury, whereas 400 μM was selected to investigate the non-linear response observed at high cisplatin exposure. The cochlear explants were incubated in 5% CO_2_ and at 37 °C in a humidified atmosphere [19].

**F-actin labeling and terminal deoxynucleotidyl transferase biotin-dUTP nick-end labeling (TUNEL) staining**

After culturing with cisplatin for 48 h, the cochlear explants were fixed for 1 h in 4% paraformaldehyde at room temperature and subsequently rinsed with 0.1 M phosphate-buffered saline (PBS). The explants were blocked with 5% horse serum and 0.5% Triton X-100 for 1 h at room temperature. To visualize F-actin that is heavily expressed in the cuticular plate and stereocilia bundles of HCs, the specimens were immersed in rhodamine-labeled phalloidin (0.5 µg/mL, P1951, Sigma, St. Louis, MO, USA) for 45 min at room temperature. After rinsing with 0.1 M phosphate-buffered saline containing 0.05% Tween 20 (PBST), specimens were mounted on glass slides in AntiFade Reagent (with DAPI) (G1407, Servicebio, Wuhan, China) and examined under a laser scanning confocal microscope (LEICA, TSC SP8) with the appropriate filter. Confocal images were processed using Confocal Assistant and Adobe Photoshop CS6 software. A hair cell was considered missing if the stereocilia or cuticular plate were not seen. The three rows of OHC and a single row of IHC for each 0.24 mm segment were counted over the entire length of the organ of Corti from apex to base. Individual cochleograms were conducted to show the percentage of IHC and OHC loss as a function of percent distance from the apex to the base. The individual cochleograms in each group were averaged to obtain mean cochleograms.

To detect apoptosis of the HCs, the cochlear explants treated with 10 µM cisplatin for 0, 24, 48, and 72 h were evaluated for TUNEL staining using Roche In Situ Cell Death TMR Red Kit (12156792910, Roche, Basel, Switzerland), according to the manufacturer’s protocol. The other cochlear explants were treated with different concentrations (0, 10, 100, and 400 μM). The specimens were fixed with 4% paraformaldehyde. Afterward, the tissues were rinsed in PBS and permeabilized with 0.5% Triton X-100 in PBS for 1 h. The cochlear explants were then incubated in a 50 µL TUNEL reaction mixture, which contained 5 µL of enzyme solution and 45 µL of label solution, as part of the terminal deoxynucleotidyl transferase-mediated dUTP-biotin nick-end labeling (TUNEL) assay (C1086, Beyotime, Shanghai, China). This incubation occurred for 60 min in the dark at a temperature of 37 °C, following the manufacturer’s instructions. After rinsing with PBST, the samples were labeled with fluorescein isothiocyanate phalloidin, as described above, and then examined under a laser scanning confocal microscope (LEICA, TSC SP8, GER).

**Spiral ganglion neurons immunofluorescence staining**

After being cultured for 48 h, the cochlear explants were fixed for 1 h in 4% paraformaldehyde at room temperature. The explants were thoroughly washed with PBS and blocked with 5% horse serum and 0.5% Triton X-100 for 1 h. Then explants were immunostained for 24 h at 4 °C with the primary antibody against TUJ1 (1:200, 801202, BioLegend, San Diego, CA, USA), which is found to especially label the neurofilaments and SGNs in cochlear and against Caspase-3 (1:200; 9668T, Cell Signaling Technology, Danvers, MA, USA). The explants were rinsed with PBST and subsequently incubated for 1.5 h with secondary antibodies (AlexaFluor 488 1:200, A21202, Invitrogen, Carlsbad, CA, USA, and AlexaFluor 594 1:200, A32740, Invitrogen, Carlsbad, CA, USA). After three washes at 20 min each, the explants were mounted in AntiFade Reagent and examined by a laser scanning confocal microscope (LEICA, TSC SP8, GER).

**Transmission Electron Microscopy (TEM)**

The cochlear explants exposed to various doses of cisplatin (control, 10 µM, 100 µM, and 400 µM) for 48 h were harvested and rinsed three times with 0.1 M phosphate buffer (PB) at 4 °C. The specimens were fixed with 2.5% glutaraldehyde for 2 h and then rinsed three times with PB, and fixed against with 1% osmic acid for 2 h. After rinsing with PB, the specimens were immersed in ascending concentrations of alcohol. The dehydrated tissues were embedded in acetone-embedding medium (2:1) at room temperature for 3 h, followed by acetone-embedding medium (1:2) at room temperature overnight, and embedding medium at 37 °C for 2 h. The embedded blocks were cured at 37 °C overnight and then at 45 °C for 12 h and 60 °C for 24 h. Afterward, blocks were cut at 16 µm thickness, and the slices were stained with 3% uranyl acetate-lead citrate and examined using transmission electron microscopy (JEOL, JEM-1230, Tokyo, Japan).

**Western blot (WB) analysis**

After treatment with various doses of cisplatin (control, 10 µM, 100 µM, and 400 µM) for 48 h, the cochlear samples were prepared by extracting proteins with Radio-Immunoprecipitation Assay (RIPA) buffer (P0013B, Beyotime, Shanghai, China) on ice, enriched with 1% phenylmethylsulfonyl fluoride, 2% protease inhibitors, and 2% phosphatase inhibitors to prevent degradation. Protein concentrations were determined using a BCA protein assay, and equal amounts of total protein (30 μg per lane) were loaded in each lane for SDS-PAGE. The protein samples clarified by centrifugation after lysis were separated by sodium dodecyl sulfate–polyacrylamide gel electrophoresis (SDS-PAGE) and electroblotted onto polyvinylidene difluoride (PVDF) membranes. Subsequently, non-specific binding sites on the membranes were occluded with a 5% skim milk solution for 1.5 h at room temperature, followed by overnight incubation at 4 °C with the following primary antibodies: anti-PTEN (1:1000, 9559S, Cell Signaling Technology, Danvers, MA, USA), anti-PI3K (1:1000, 4257T, Cell Signaling Technology, Danvers, MA, USA), anti-AKT (1:1000, 4691T, Cell Signaling Technology, Danvers, MA, USA), anti-Phospho-AKT (1:1000, Cell Signaling Technology,4060T, Danvers, MA, USA), anti-Parkin (1:1000, 66674-1, Proteintech, Rosemont, IL, USA), anti-P62 (1:1000, GB11531, Servicebio, Wuhan, China), anti-cleaved caspase 3 (1:1000, 9664, Cell Signaling Technology, Danvers, MA, USA), and anti-β-ACTIN (1:3000, 4970, Cell Signaling Technology, Danvers, MA, USA). After primary antibody incubation, the membranes were incubated for 2 h with horseradish peroxidase-conjugated secondary antibodies. Protein bands were detected using an enhanced chemiluminescence detection reagent, Bio-Rad ChemiDoc XRS+ (Bio-Rad Company, Hercules, CA, USA), and the band intensities were quantified using ImageJ (version 1.53) software.

**Quantitative PCR Array**

The total RNA was isolated from basilar membrane explants following 48 h of cisplatin exposure using TRIzol reagent (15596018, Invitrogen, Carlsbad, CA, USA). The RNA was purified by chloroform extraction and isopropanol precipitation, washed with 75% ethanol, air-dried, and resuspended in DEPC-treated water. Genomic DNA was removed prior to cDNA synthesis using the PrimeScript RT Reagent Kit with gDNA Eraser (RR047A, Takara Bio, Kusatsu, Shiga, Japan). Quantitative real-time PCR was performed using commercial PCR arrays targeting GABA/glutamate signaling and autophagy-related genes (wc-mRNA0060-M, wc-mRNA0268-M, WCGENE BIOTECH, Shanghai, China). Amplification was carried out with an initial denaturation at 95 °C for 3 min, followed by 40 cycles at 95 °C for 10 s and 60 °C for 30 s, and melt curve analysis was performed thereafter. Relative mRNA levels were determined using the 2^−ΔΔCt^ method after normalization to housekeeping genes, with the control group serving as the reference. The data were analyzed using Wcgene Biotech software (WCGene Biotech, Shanghai, China) Genes with fold-changes greater than or less than 2.0 were considered to be of biological significance.

**Statistical analysis**

The investigators were aware of group allocation during the treatment and experimental procedures. The outcome assessment, including hair cell counting, fluorescence-image quantification, and other scoring procedures, was performed blinded to group allocation. Group identities were revealed only after completion of data analysis. Statistical evaluations were performed utilizing GraphPad Prism (version 9.0) software. All data were depicted as the mean ± standard deviation (SD), ensuring reliability through the repetition of each experiment a minimum of three times. We applied an unpaired Student’s *t*-test to assess differences between the two groups. For analyses involving multiple groups (more than two), a one-way ANOVA was conducted, followed by a Dunnett multiple comparison test. A significance level of *p* < 0.05 was set to identify statistically significant outcomes.

## 3. Results

### 3.1. Non-Linear Hair Cell Injury Induced by Cisplatin

**Figure 1:**
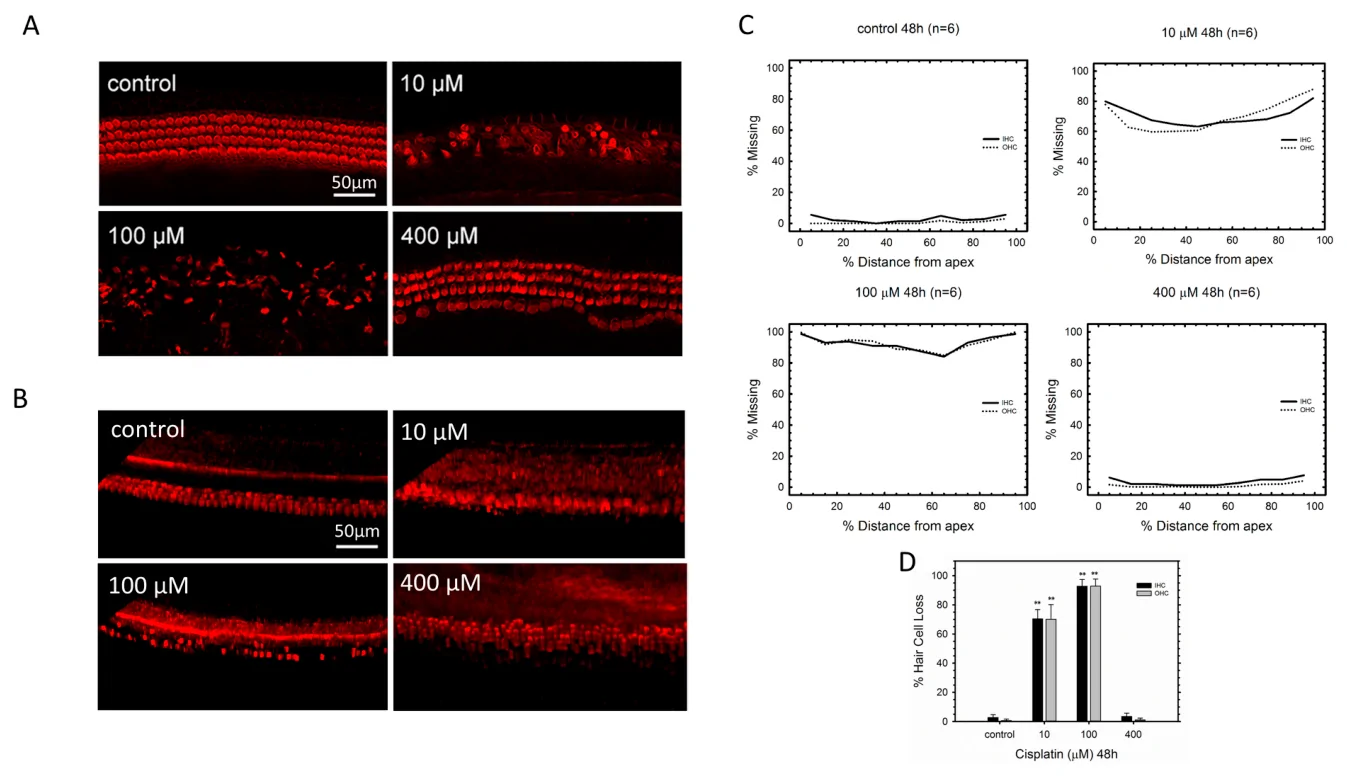
Cisplatin-induced hair cell damage. (**A**) Representative images showing the number of HCs in the different groups treated with cisplatin (0, 10, 100, and 400 µM); n represents independent cochlear explants, *n* = 6. (**B**) Representative 3D images; n represents independent cochlear explants, n = 6. (**C**) An illustration of the percent missing of IHCs and OHCs with cisplatin (0 µM); n represents independent cochlear explants, n = 6. (**D**) A comparison of the percent loss of IHCs and OHCs in the different groups. ** *p* < 0.01 vs. the control group. Scale bars: 50 μm.

Examination of the organ of Corti following 48 h of cisplatin exposure at various concentrations (10, 100, 400 µM) revealed that cisplatin-induced ototoxicity did not appear in a dose-dependent manner. It showed the orderly arrangement of the cochlea’s three rows of outer hair cells (OHCs) and one row of inner hair cells (IHCs) in the control cochlear cultures. The cochlear explants treated with 10 µM cisplatin had partial or complete loss of stereocilia bundles on some HCs and disorderly rows of IHCs and OHCs. Few residual HCs were observed when the cochlear explants were exposed to 100 µM cisplatin. However, when the concentration of cisplatin was increased to 400 µM, HCs were nearly the same as normal control cultures (Figure 1A,B).

To evaluate the location and degree of cochlear pathology, mean cochleograms were produced, showing the percent missing of IHCs and OHCs as a function of the percent distance from the apex of the cochlea to the base for each cisplatin concentration. The in vitro culture of cochlear HCs in the control group grew well during the 48 h culture, and only an occasional inner or outer loss of HCs occurred. The 10 µM cisplatin caused approximately 70% of IHCs and 71% of OHCs to disappear, with statistically significant loss in both two types of HCs compared to the normal control group (*p* < 0.01). When the concentration of cisplatin was increased to 100 µM, there was a significant loss of IHCs and OHCs in contrast to the control group (*p* < 0.01). Only about 10% of IHCs and OHCs were observed at the 100 µM concentration (Figure 1C). There was little IHC or OHC loss in cochlear cultures treated with 400 µM cisplatin, which was not a statistically significant increase in IHC or OHC loss in contrast to the control group (*p* > 0.05) (Figure 1C,D). These results showed that cisplatin destroyed HCs and there was no obvious difference between the two kinds of HCs in terms of the loss rate. Cisplatin-induced HC injury showed a non-linear concentration–response pattern at the 48 h time point, with substantially less HC loss at 400 μM than at 100 μM. This difference reflects the extent of HC loss rather than the function of the remaining cells alone [20]. However, delayed toxicity at 400 μM cannot be excluded.

### 3.2. Reduced HC Apoptosis at High Cisplatin Concentrations

**Figure 2:**
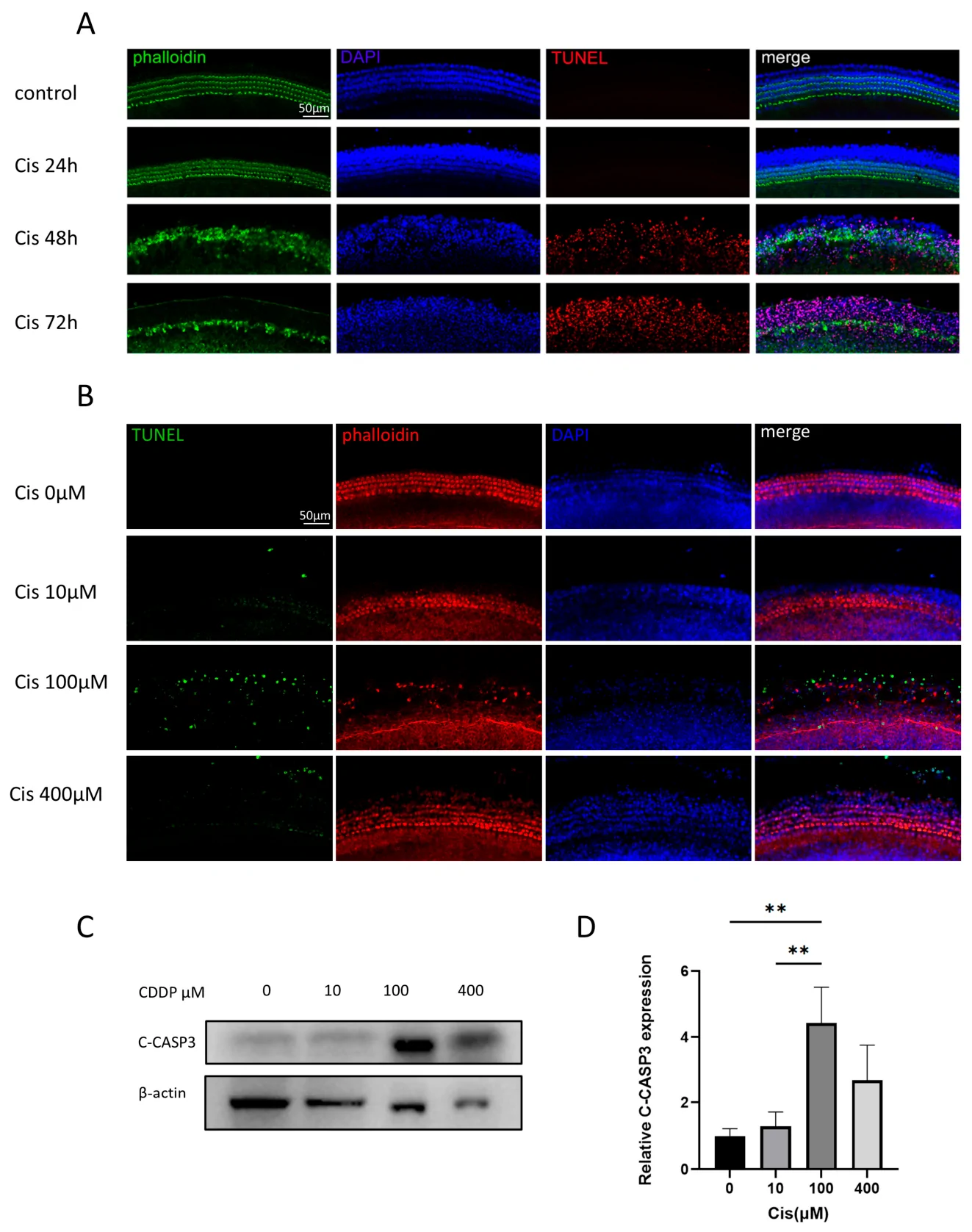
Cisplatin-induced hair cell apoptosis. (**A**) Phalloidin (green), TUNEL (red), and DAPI (blue) staining showed the number of apoptotic cells in the different groups treated with cisplatin (0, 24, 48, and 72 h); n represents independent cochlear explants, n = 3. (**B**) TUNEL (green), Phalloidin (red), and DAPI (blue) staining showed the number of apoptotic cells in the different groups treated with cisplatin (0, 10, 100, and 400 µM). Scale bars: 20 μm; n represents independent cochlear explants, n = 3. (**C**) Western blot results showing the expression of C-CASP3 after cisplatin treatment for 48 h; n represents independent biological experiments, n = 3. Quantitative analysis of the data shown in (**D**). ** *p* < 0.01.

To detect whether the hair cell death was mediated by apoptosis, we treated the cochlear cultures for 24 h, 48 h, and 72 h with 10 µM cisplatin, a concentration that caused a significant number of HCs to be partially but not completely missing. The explants were, respectively, labeled with Alexa 488-phalloidin, 4′,6-diamidino-2-phenylindole (DAPI), and TUNEL. The HCs and their nuclei were arranged in orderly rows in the control group. The nuclei of the Hensen cells could also be seen near the third row of the OHCs. These nuclei were round and similar in size. Apoptosis-positive nuclei were not seen in the control group. When the explants were incubated with 10 µM cisplatin for 24 h, the HCs and their nuclei were also aligned in orderly rows and were of regular size. TUNEL-positive cells were not observed. Interestingly, much more nuclei of supporting cells were detected. After the cochlear explants were treated with 10 µM cisplatin for 48 h, many TUNEL-positive cells were observed in the HCs and supporting cells region. Most of the HCs were destroyed and their nuclei were condensed, a hallmark of apoptosis. In addition, the nuclei of some supporting cells showed obvious morphological changes in apoptosis. When treated with 10 µM cisplatin for 72 h, only a few HCs were disorganized. The nuclei in the HCs and supporting cells region tended to be highly condensed. TUNEL-positive cells were widespread throughout the whole explants (Figure 2A). These results showed that 10 µM cisplatin induced apoptosis in HCs and supporting cells in a time-dependent manner.

To further investigate the mechanisms underlying the attenuation of cisplatin-induced damage to HCs in cochlear explants cultured in vitro, we exposed the cochlear cultures to various concentrations (10, 100, and 400 µM) of cisplatin for 48 h. The results from the TUNEL assay demonstrated that in the control group, the HCs were neatly arranged with regular nuclear morphology and orderly arrangement, with no apoptotic signals detected. After 48 h of exposure to 10 µM cisplatin, both the IHCs and OHCs exhibited disordered structural arrangement and a significant loss of HCs, and most hair cell nuclei were shrunken and fragmented. The nuclei of adjacent supporting cells remained relatively regular. The TUNEL assay detected a large number of apoptotic signals in the hair cell region. Following 48 h of exposure to 100 µM cisplatin, the toxic damage was further exacerbated, leaving only a few HCs in the basilar membrane. Extensive nuclear fragmentation occurred, and adjacent supporting cells also exhibited notable nuclear fragmentation. The TUNEL assay revealed that the hair cell region was almost entirely composed of apoptotic signals. However, after 48 h of exposure to 400 µM cisplatin, the cytotoxic damage was relatively reduced compared to the 100 µM cisplatin treatment group, and the TUNEL assay detected a corresponding decrease in apoptotic signals in the hair cell region (Figure 2B).

Consistent with the TUNEL immunofluorescence results, WB analysis revealed a marked increase in cleaved caspase-3 (C-CASP3) expression after 48 h of exposure to 100 µM cisplatin compared to the higher concentration of 400 µM cisplatin and the control group (Figure 2C,D). This suggests that in the low-concentration cisplatin group, cochlear explant damage was due to apoptosis, whereas in the high-concentration cisplatin group, the damage to the cochlear explant was alleviated and the degree of apoptosis was also reduced.

### 3.3. Reduced SGN Injury at High Cisplatin Concentration

**Figure 3:**
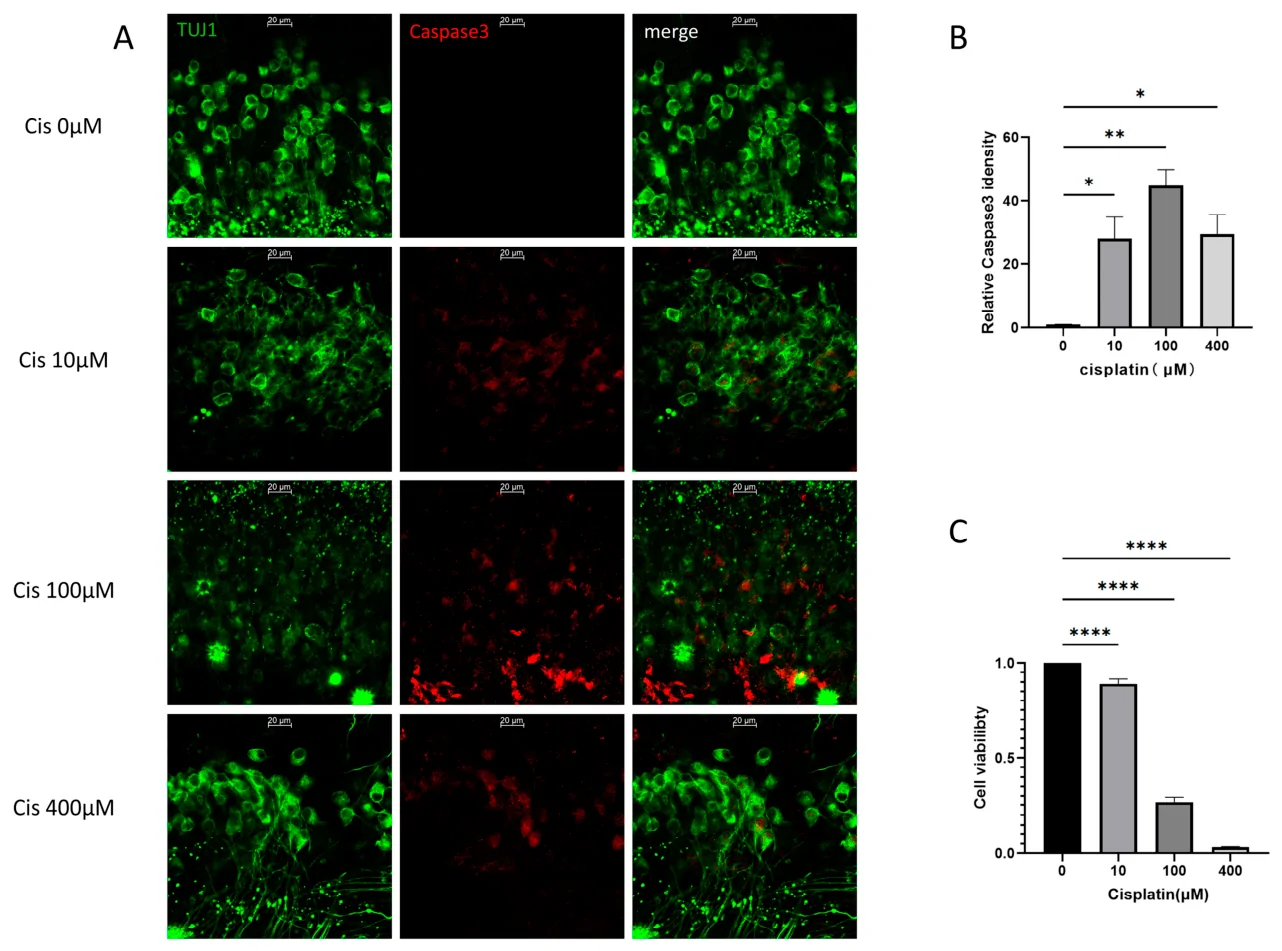
Cisplatin destroyed the SGNs and nerve fibers and induced apoptosis. (**A**) C-CASP3 (red) and TUJ1 (green) double staining showed that the expression of apoptotic cells was significantly decreased after the cisplatin treatment (400 µM) compared with the group treated with cisplatin (100 µM); n represents independent cochlear explants, n = 3. (**B**) Quantitative analysis of the C-CASP3-positive cells. (**C**) CCK-8 assay showing the viability of the HEI-OC1 cells after treatment with cisplatin (0, 10, 100, and 400 µM); n represents independent biological experiments, n = 3. * *p* < 0.05, ** *p* < 0.01, and **** *p* < 0.0001 vs. the control group. Scale bars: 20 μm.

The spiral ganglion neurons (SGNs) are also primary targets of cisplatin-induced ototoxicity within the inner ear. Subsequently, TUJ1 and cleaved caspase-3 immunostaining were performed to investigate SGN morphology and apoptosis following exposure to different concentrations of cisplatin. In the control group, the SGNs and auditory nerve fibers (ANFs) exhibited preserved morphology with minimal cleaved caspase-3 staining. After 48 h of exposure to 10 μM cisplatin, the SGN morphology was disrupted, and the ANFs appeared distorted. More severe structural damage was observed after 100 μM cisplatin treatment, with fewer morphologically preserved SGNs and ANFs and increased cleaved caspase-3-positive staining. However, when treated with 400 μM cisplatin, the cochlear explants exhibited more preserved SGN and ANF morphology and fewer cleaved caspase-3-positive cells than the 100 μM group (Figure 3A). Quantification of the cleaved caspase-3-positive cells confirmed reduced apoptosis in the 400 μM group compared with the 100 μM group (Figure 3B). These findings indicate that SGN injury and apoptosis were less pronounced at 400 μM than at 100 μM cisplatin.

Subsequently, we treated the House Ear Institute Organ of Corti 1 (HEI-OC1) cells with varying concentrations of cisplatin (0, 10, 100, and 400 μM) for 48 h to determine whether the non-linear response observed in the cochlear explants was reproduced in the cell-line model. The results indicated that HEI-OC1 cell viability declined progressively with increasing cisplatin concentration. The survival rate of HEI-OC1 cells was reduced to less than 10% following 48 h of treatment with 400 μM cisplatin (Figure 3C). Thus, the non-linear response observed in the cochlear explants was not reproduced in HEI-OC1 cells.

### 3.4. Cisplatin-Induced Changes in Neurotransmission- and Autophagy-Related Genes

**Figure 4:**
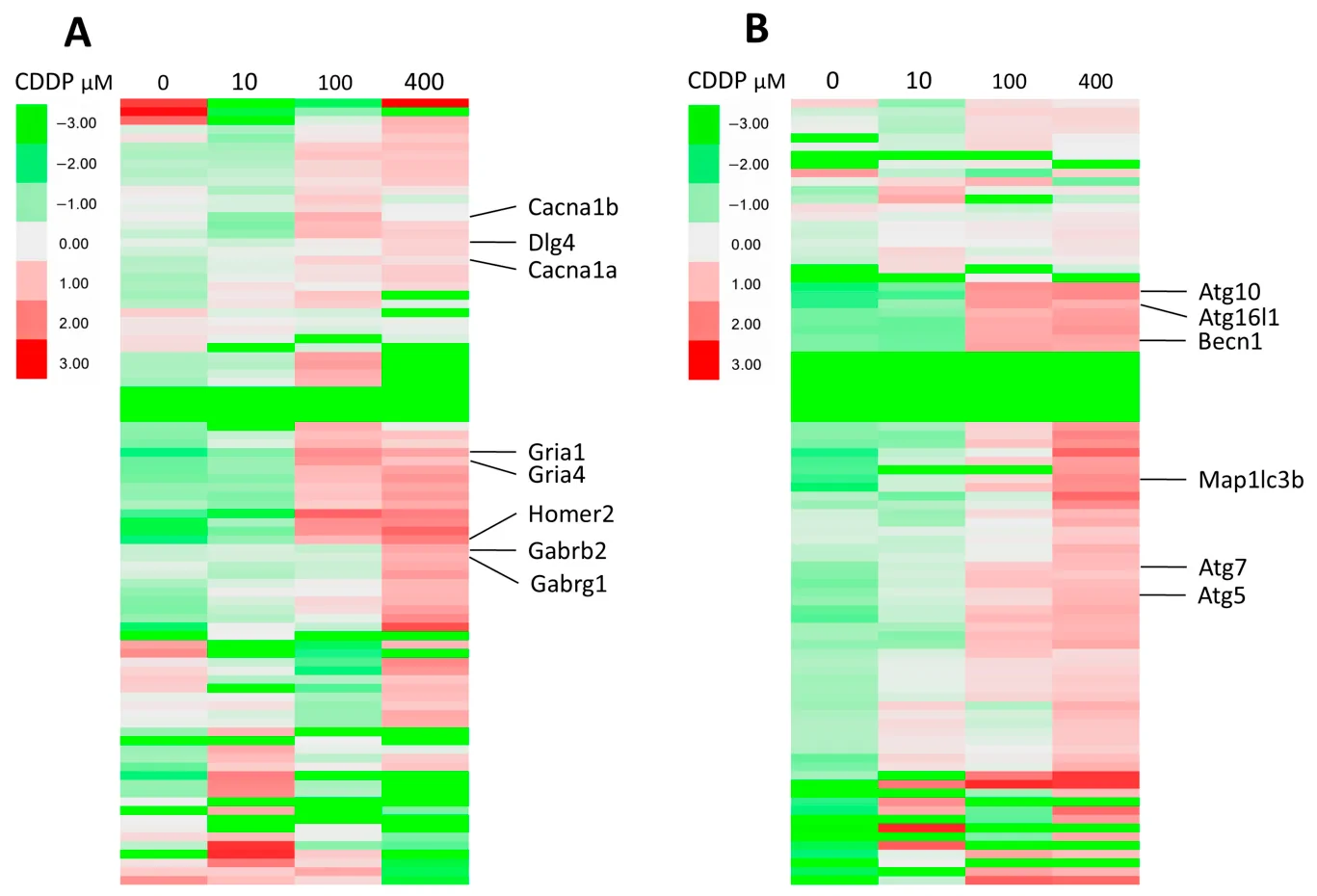
PCR array analysis of the cochlear explants after 48 h of culture in the indicated groups. The data are shown as Z-scores. (**A**) GABA and glutamate signaling pathway-related factors. (**B**) Autophagy signaling pathway-related factors; n represents independent biological experiments, n = 3.

To investigate the molecular changes associated with the non-linear response to cisplatin, PCR array analyses were performed to assess neurotransmission- and autophagy-related gene expression. Compared with the control group, 100 μM cisplatin increased the expression of glutamatergic signaling-related genes, including Gria1 and Gria4, as well as the calcium channel-related genes Cacna1a and Cacna1b. At 400 μM cisplatin, Gria1 and Gria4 expression was lower than that observed at 100 μM, while Cacna1b expression approached the control level. In addition, Dlg4, Homer2, Gabrg1, and Gabrb2, which are associated with synaptic organization and GABAergic signaling, were upregulated in the 400 μM group (Figure 4A).

Cisplatin exposure also altered the expression of autophagy-related genes. Compared with the control group, 100 μM cisplatin increased the expression of Atg5, Atg7, Atg10, Atg16l1, Becn1, and Map1lc3b. The expression of Map1lc3b, Atg16l2, Sqstm1, and Lamp3 was increased in the 400 μM group. Moreover, compared with the 100 μM group, 400 μM cisplatin decreased Akt1 expression and increased Pik3cg expression (Figure 4B). Collectively, these results demonstrate distinct concentration-dependent transcriptional changes in genes associated with neurotransmission, calcium signaling, synaptic organization, autophagy, and PI3K/AKT signaling. These findings provide exploratory pathway-level evidence and were not used to establish functional changes in these pathways.

### 3.5. Enhanced Autophagy and PTEN/PI3K/AKT Signaling at High Cisplatin Concentrations

Based on the above results, 10 µM and 100 µM cisplatin caused important ototoxicity. However, 400 μM cisplatin treatment resulted in near-normal HCs and SGNs appearance and reduced ototoxicity compared with that of lower concentrations. It seemed that 400 µM cisplatin induced resistance in the HCs and SGNs. To investigate the molecular mechanisms underlying the protective effects of cisplatin at high concentrations in reducing damage to the HCs and SGNs, TEM and WB were performed to explore whether this cisplatin resistance is mediated by autophagy. We treated cochlear explants with 10 µM and 400 µM cisplatin for 48 h and evaluated the status of the HCs and SGNs.

**Figure 5:**
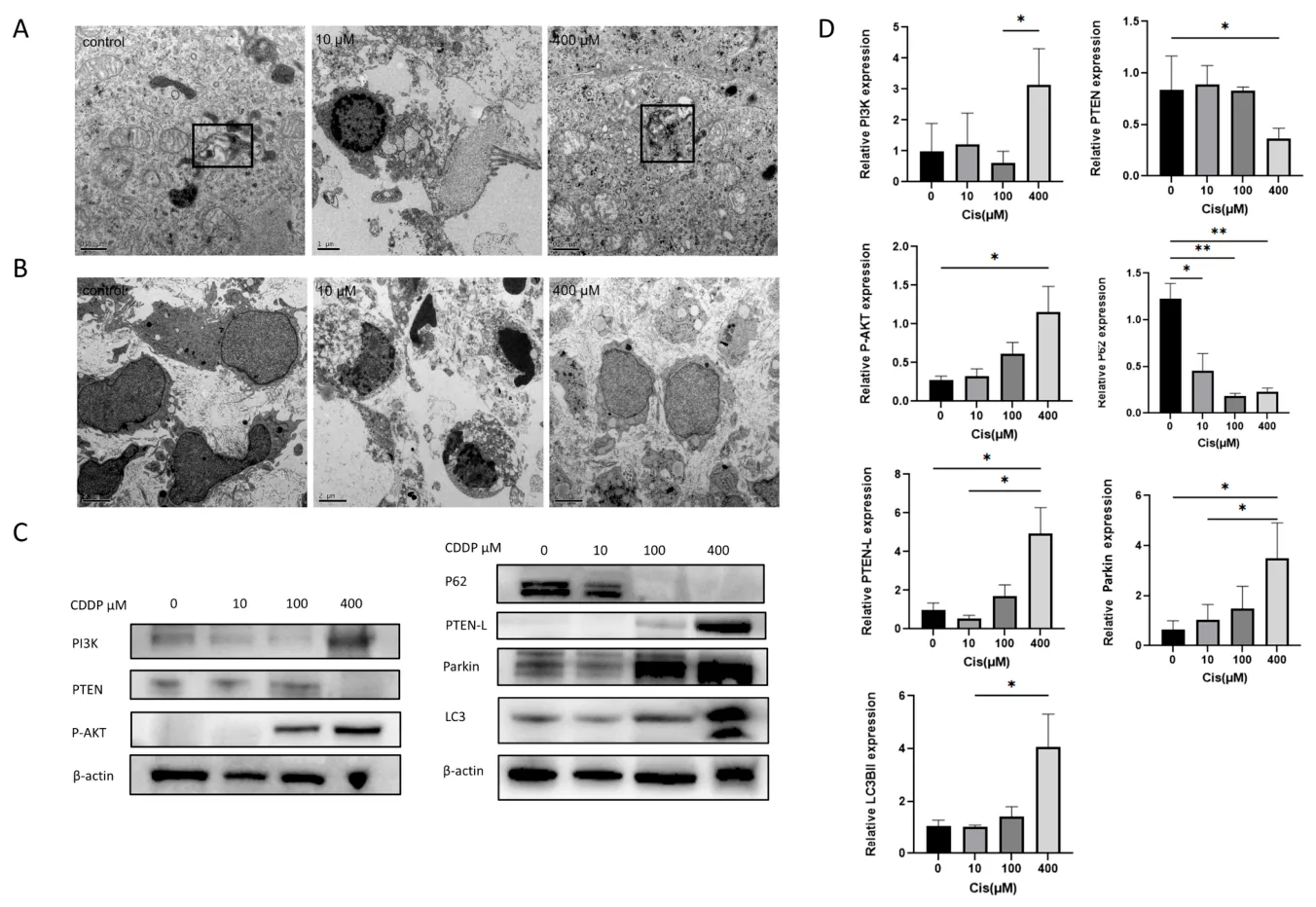
Autophagy and PTEN/PI3K/AKT signaling following cisplatin exposure. The cochlear explants from the P3 SD WT Rat pups were cultured and incubated with cisplatin for 48 h. (**A**) TEM analysis evaluated autophagy in the HCs; n represents independent cochlear explants, n = 4. Autolysosomes are seen in the cytoplasm of the outer hair cell in both the control group and the 400 µM cisplatin group. Significant apoptosis was seen in the 10 µM cisplatin group. Scale bars: 0.5 μm (control and 100 μM) and 1 μm (10 μM). (**B**) TEM analysis evaluated autophagy in the SGNs; n represents independent cochlear explants, n = 4. Scale bars = 2 μm. (**C**) Western blot results showing the expression of P62, PTEN-L, Parkin, LC3II, PI3K, PTEN, and P-AKT after cisplatin treatment for 48 h in the different groups; n represents independent biological experiments, n = 3. (**D**) Quantitative analysis of the data shown in (**C**): * *p* < 0.05 and ** *p* < 0.01.

TEM analyses revealed that the cellular morphology of the HCs in the 10 µM cisplatin treatment group displayed apoptotic characteristics compared to the control group. These characteristics included hair cell swelling, disruption of the cell membrane, condensation of nuclear chromatin, and fragmentation of organelles. The organization of the HCs was irregular, and the internal cellular architecture was significantly compromised. In contrast, the group subjected to 400 µM cisplatin exhibited no notable alterations in nuclear structure. However, mild swelling of the mitochondria was observed, along with the presence of autophagic structures encased by double membranes. The control group showed no significant changes in the nuclei of the HCs, no notable mitochondrial swelling, and only sporadic observations of autophagic structures enveloped by double membranes (Figure 5A). Following 48 h of in vitro culture, the control group exhibited the clearly defined contours of the SGNs, a uniform nuclear gray level, a high nucleus-to-cytoplasm ratio, and no significant abnormalities were detected in the organelles. In the 10 µM cisplatin group, the cellular outlines were blurred, with chromatin within the nucleus becoming condensed, an increase in gray levels, and organelles exhibiting pronounced ballooning, indicative of cell apoptosis. Conversely, in the 400 µM cisplatin group, the cell outlines remained well-defined. Furthermore, the nuclei displayed a uniform grayscale intensity and an elevated nucleus-to-cytoplasm ratio, while the cellular outlines continued to be distinctly recognizable. Lipid droplets were commonly noted in the cytoplasm, whereas the cellular organelles did not exhibit significant swelling (Figure 5B).

Incorporating the previously mentioned TEM findings, the ultrastructural alterations in the basilar membrane following 48 h of exposure to different concentrations of cisplatin aligned with the morphological assessments. Specifically, the ototoxic effects of cisplatin did not intensify with higher concentrations. Low concentrations (10 µM) of cisplatin were observed to trigger apoptosis in the HCs and spiral ganglion neurons, a finding that was further corroborated by the TEM analysis. Conversely, high concentrations (400 µM) of cisplatin caused some damage to intracellular organelles in the HCs and spiral ganglion neurons. However, their overall morphological integrity remained intact. Autophagic structures were identified within the HCs in both the high-concentration and control groups, indicating potential cell survival. In contrast, the activation of apoptosis in the absence of autophagic structures was observed in the HCs of the low-concentration group, leading to cell death. It is postulated that autophagy may be involved in the mechanisms of action of cisplatin at varying concentrations.

To further investigate whether autophagy is involved in the protective effects of high concentrations of cisplatin, WB was employed to assess the expression of the autophagy protein. The level of Microtubule-associated protein 1A/1B-light chain 3 II (LC3II) increased with higher concentrations of cisplatin treatment, while Sequestosome 1 (SQSTM1/p62) levels decreased. The increase in LC3-II, decrease in p62, and presence of autophagic structures were consistent with enhanced autophagy-associated activity at higher cisplatin concentrations. However, autophagic flux was not directly assessed. To determine which upstream pathway leads to increased autophagy, the protein levels of PTEN were examined using WB. The results indicated that PTEN levels were reduced, while the levels of PI3K and phosphorylated AKT (p-AKT) increased in the high concentrations of cisplatin group (400 µM) compared to both the low-concentration cisplatin group (10 and 100 µM) and the control group (Figure 5C,D). Taken together, the reduced ototoxicity of cisplatin at a high concentration may be attributed to the activation of the PTEN-AKT signaling pathway, which enhances autophagy in the HCs and SGNs.

PINK1-Parkin-mediated mitophagy is a key mechanism of mitochondrial quality control and has been implicated in cochlear injury. Recent studies suggest that PTEN-L negatively regulates this pathway. Notably, both Parkin and PTEN-L were elevated in the 400 µM cisplatin group (Figure 5C,D), suggesting that mitochondrial quality-control signaling may be involved in the response to high-concentration cisplatin.

## 4. Discussion

The reduced injury at 400 μM was accompanied by decreased apoptosis and enhanced autophagy-associated activity, as reflected by increased LC3II, decreased P62, and autophagic structures observed by transmission electron microscopy. Non-monotonic dose–response relationships have been reported in toxicological systems and may arise from opposing biological processes [21]. The lower cochlear injury at 400 μM than at 100 μM cisplatin is consistent with such a pattern. Previous studies have reported both protective and detrimental roles of autophagy in ototoxicity. In several cisplatin- and aminoglycoside-induced models, enhanced autophagy was associated with reduced cochlear injury, whereas excessive or impaired autophagic processing has also been linked to cellular damage [22,23]. Our findings are, therefore, more consistent with a context-dependent role of autophagy. Nevertheless, autophagy-related changes were already present at 100 μM, where injury remained severe, indicating that autophagy alone is unlikely to determine cochlear cell fate. Moreover, because autophagic flux was not directly assessed using lysosomal inhibitors, these findings support enhanced autophagy-associated activity rather than definitively increased autophagic flux.

PTEN/PI3K/AKT signaling was also altered at 400 μM, with decreased PTEN and increased PI3K and phosphorylated AKT. Although activation of the canonical PI3K/AKT/mTOR pathway generally suppresses autophagy [24], AKT activation and enhanced autophagy may coexist under specific stress conditions through parallel, compensatory, or mTOR-independent mechanisms [17,18]. Thus, the concurrent changes observed here should not be interpreted as evidence that PI3K/AKT activation directly promotes autophagy. Similarly, the concomitant increase in Parkin and PTEN-L may reflect remodeling of mitochondrial quality-control signaling rather than direct evidence of enhanced mitophagy [25,26,27,28]. Taken together, reduced apoptosis at 400 μM occurred together with enhanced autophagy-associated activity and altered PTEN/PI3K/AKT and mitochondrial quality-control signaling, which may collectively contribute to the reduced tissue injury. Functional inhibition and flux experiments will be required to clarify these relationships.

Importantly, the response was observed in the cochlear explants but not in the HEI-OC1 cells, in which viability declined progressively with increasing cisplatin concentrations, suggesting that the phenomenon is model-dependent. Under the standard 33 °C culture condition, the HEI-OC1 cells remained proliferative and relatively undifferentiated, limiting direct comparison with mature cochlear hair cells. Unlike the isolated HEI-OC1 cells, the cochlear explants preserved interactions among the HCs, supporting cells, SGNs, and the surrounding tissue microenvironment, which may modify cellular stress responses to cisplatin. In addition, only a limited number of concentrations and primarily a 48 h time point were examined, so delayed toxicity at 400 μM and the precise shape of the dose–response curve remain unresolved. This concentration was used as a severe stress condition to investigate the previously observed non-linear response and should not be interpreted as representing a clinically achievable cochlear cisplatin concentration. Although equal amounts of total protein were loaded for Western blotting, the overall protein yield from the cochlear explants decreased at higher cisplatin concentrations. Therefore, the Western blot results represent relative protein abundance under equal-protein loading conditions, rather than absolute protein abundance per explant. The reduced total protein recovery at high cisplatin concentrations also indicates that preserved morphology does not necessarily imply preserved cellular function.

In addition, oxidative stress biomarkers were not directly assessed, limiting our ability to determine how ROS-related mechanisms contribute to the non-linear response [29,30]. The PCR array further suggested coordinated transcriptional alterations in neurotransmission- and autophagy-related pathways. However, these findings should be considered exploratory, pathway-level evidence, as selected targets were not independently validated by qRT-PCR or Western blotting. Therefore, the present findings support an association among reduced cochlear injury, decreased apoptosis, enhanced autophagy-associated activity, and altered PTEN/PI3K/AKT signaling, rather than establishing a causal protective mechanism.

## 5. Conclusions

Cisplatin induced a non-linear ototoxic response in cochlear organotypic cultures. The reduced HC and SGN injury observed at 400 μM was accompanied by decreased apoptosis, enhanced autophagy-associated activity, and altered PTEN/PI3K/AKT signaling, consistent with a tissue-level adaptive stress response. This response was not reproduced in HEI-OC1 cells, highlighting the potential importance of the multicellular cochlear microenvironment. Further studies using pharmacological or genetic inhibition of autophagy and PI3K/AKT signaling are required to determine the causal mechanisms underlying this non-linear response.

## Data Availability

The data supporting the findings of this study are available from the corresponding author upon reasonable request.

## Abbreviations

The following abbreviations are used in this manuscript:

HCs	Hair cells
SGNs	Spiral ganglion neurons
HEI-OC1	House Ear Institute–Organ of Corti 1
qPCR	Quantitative PCR
PI3K	Phosphatidylinositide 3-kinases
AKT	Protein kinase B
LC3II	Microtubule-associated protein 1 light chain 3 II
STS	Sodium thiosulfate
ROS	Reactive oxygen species
miR	MicroRNA
Bcl-2	B-cell lymphoma-2
XIAP	X-linked inhibitor of apoptosis protein X
PRDX1	Peroxiredoxin 1
DMEM	Dulbecco’s modified Eagle’s medium
CCK-8	Cell Counting Kit-8
PBS	Phosphate-buffered saline
OHC	Outer hair cell
IHC	Inner hair cell
TUNEL	Terminal deoxynucleotidyl transferase dUTP nick-end labeling
TEM	Transmission Electron Microscopy
CDDP	Cisplatin
WB	Western blot
RIPA	Radio-Immunoprecipitation Assay
SDS-PAGE	Sodium dodecyl sulfate–polyacrylamide gel electrophoresis
PVDF	Polyvinylidene difluoride
SD	Standard deviation
C-CAPS3	Cleaved caspase 3
ANFs	Auditory nerve fibers
